# Effects of Donor Ages and Propagation Methods on Seedling Growth of *Platycladus orientalis* (L.) Franco in Winter

**DOI:** 10.3390/ijms24087170

**Published:** 2023-04-12

**Authors:** Yao Dong, Wenfa Xiao, Wei Guo, Yifu Liu, Wen Nie, Ruizhi Huang, Cancan Tan, Zirui Jia, Jianfeng Liu, Zeping Jiang, Ermei Chang

**Affiliations:** 1 Key Laboratory of Forest Ecology and Environment of National Forestry and Grass-Land Administration, Ecology and Nature Conservation Institute, Chinese Academy of Forestry, Beijing 100091, China; 2 Taishan Academy of Forestry Sciences, Taian 271000, China; 3 State Key Laboratory of Tree Genetics and Breeding, Key Laboratory Tree Breeding and Cultivation of the National Forestry and Grassland Administration, Research Institute of Forestry, Chinese Academy of Forestry, Beijing 100091, China

**Keywords:** arborvitae, ancient trees, propagation methods, low temperature, transcriptome

## Abstract

To evaluate the effects of donor ages on growth and stress resistance of 6-year-old seedlings propagated from 5-, 2000-, and 3000-year-old *Platycladus orientalis* donors with grafting, cutting, and seed sowing, growth indicators and physiological and transcriptomic analyses were performed in 6-year-old seedlings in winter. Results showed that basal stem diameters and plant heights of seedlings of the three propagation methods decreased with the age of the donors, and the sown seedlings were the thickest and tallest. The contents of soluble sugar, chlorophyll, and free fatty acid in apical leaves of the three propagation methods were negatively correlated with donor ages in winter, while the opposite was true for flavonoid and total phenolic. The contents of flavonoid, total phenolic, and free fatty acid in cutting seedlings were highest in the seedlings propagated in the three methods in winter. KEGG (Kyoto Encyclopedia of Genes and Genomes) enrichment analysis of differentially expressed genes showed phenylpropanoid biosynthesis and fatty acid metabolism pathways, and their expression levels were up-regulated in apical leaves from 6-year-old seedlings propagated from 3000-year-old *P. orientalis* donors. In addition, hub genes analysis presented that *C4H*, *OMT1*, *CCR2*, *PAL*, *PRX52*, *ACP1*, *AtPDAT2*, and *FAD3* were up-regulated in cutting seedlings, and the gene expression levels decreased in seedlings propagated from 2000- and 3000-year-old donors. These findings demonstrate the resistance stability of cuttings of *P. orientalis* and provide insights into the regulatory mechanisms of seedlings of *P. orientalis* propagated from donors at different ages in different propagation methods against low-temperature stress.

## 1. Introduction

Thousands of years of ancient trees have high natural, cultural, ecological, and scientific research values as well as gene pool significance. Along with the rapid development of the city, many ancient trees have been destroyed, and the protection of ancient and valuable trees is receiving more and more attention. *Platycladus orientalis*, a species of evergreen conifer, originated in China and is widely distributed in Asia and Europe. Ancient *P. orientalis* is a long-lived tree and is particularly rich in stress resistance, longevity, and other desirable genes [1]. However, a large proportion of the ancient trees have died gradually due principally to climatic and anthropic factors, so preservation of the genetic material of the ‘gene bank’ is of special interest [2]. There are abundant resources of ancient *P. orientalis* in China, such as Xuanyuanbai and Guajiabai in the Yellow Emperor’s Tomb in Shaanxi, but there are few studies on the mechanism of low-temperature stress with ancient cypress. Methods of propagation for trees include seed sowing, cutting, and grafting. The disadvantage of sown seedlings is that the offspring are prone to variation, and it is not easy to maintain the excellent traits of the donors. While asexual propagation of cutting and grafting can permit homogeny among individuals [3]. However, there are few studies focusing on the effects of donor age on the stress resistance of seedlings propagated in different ways when grown under low-temperature conditions.

The method of propagation affects the growth characteristics of subsequent seedlings. The 4-year-old sown seedlings of *Betula pendula* Roth. grew more vigorously than the grafted ones [4]. The sown seedlings of *Allanblackia floribunda* Oliv. were the highest, followed by grafted seedlings, and the cutting seedlings were the shortest [5]. The grafted seedlings of *Coffea canephora* Pierre ex Froehn. grew much better than the cutting seedlings in nearly all the characteristics assessed [6]. Grafting propagation enhanced the ability of *Citrullus lanatus* (Thunb.) Matsum. et Nakai to tolerate low temperatures [7]. The age of scion donors has a huge impact on the stress resistance of grafted trees. The expression of pathogen and abiotic stress resistance-related genes increased in mature branches, but the effect of donor age on the resistance of offspring due to sexual or asexual reproduction is less reported [8,9,10].

Secondary metabolites play a key role in plant defense response against the cold [11]. Flavonoid biosynthesis and phenylpropanoid biosynthesis pathways positively regulated cold stress tolerance in both of *Hevea brasiliensis* Muell. Arg. and *Fagopyrum tataricum* (L.) Gaertn. [12,13]. With an increase in donor age, the production of flavonoid compounds in the phenylpropanoid biosynthesis pathway might be adversely affected in *Ginkgo biloba* L. [14,15]. The activities of various key enzymes of the phenylpropanoid biosynthetic pathway, such as Phenylalanine ammonialyase (PAL) and Chalcone synthase (CHS), were variably regulated under abiotic stresses [16]. Moreover, unsaturated fatty acids in plants can increase the fluidity and stability of the membranes to promote cold stress tolerance. The genes encoding omega-3 fatty acid desaturase and 3-ketoacyl-ACP synthase II involved in the fatty acid synthesis pathway can improve the cold tolerance of plants under a low-temperature environment [17,18]. Fatty acid synthesis pathway genes, including Fatty acyl-ACP thioesterase A (*FATA*), Delta(8)-fatty acid desaturase 2 (*SLD2*), and Fatty acid desaturase 3 (*FAD3*), play key roles in plant cold tolerance [15]. However, there have been few reports on the role of secondary metabolites in the resistance of different ages of *P. orientalis* to low-temperature stress.

In recent years, the expression of cold-resistance-related genes in plants propagated in different methods has been analyzed at the transcriptome level. For example, 75% of the differentially expressed genes (DEGs), up-regulated in grafted seedlings of *C. lanatus* at low temperatures, were involved in protein processing, plant–pathogen interaction, and spliceosomes [7,19]. It was found that the DEGs in cutting seedlings of *Vitis vinifera* L. under low temperatures were involved in ethylene signaling, ABA (abscisic acid) signaling, *AP2*/*ERF* (APETALA2/Ethylene Responsive Factor), *WRKY* and *NAC* (N-Acetylcysteine) transcription factor gene families, and starch and sucrose metabolism pathways [20].

For obtaining the optimal preservation method, the cold tolerance of ancient *P. orientalis* seedlings preserved by different propagation methods was evaluated. We collected apical leaves from the sown seedlings, cut seedlings, and grafted seedlings propagated from 5-, 2000-, and 3000-year-old *P. orientalis* donors in winter for growth indicators, physiology determination, and transcriptome sequencing. Then, we conducted a correlation analysis among basal stem diameters, heights, and gene modules. The purpose of this study was to explore the differences in stress resistance of seedlings propagated from differently aged ancient *P. orientalis* in different methods under low temperatures and to comprehensively compare the stability and reliability of asexual propagation and sowing to provide a theoretical basis for selecting an optimal propagation method for ancient *P. orientalis*.

## 2. Results

### 2.1. Age of Donors and Propagation Methods Affect the Growth of Seedlings in Winter

**Figure 1:**
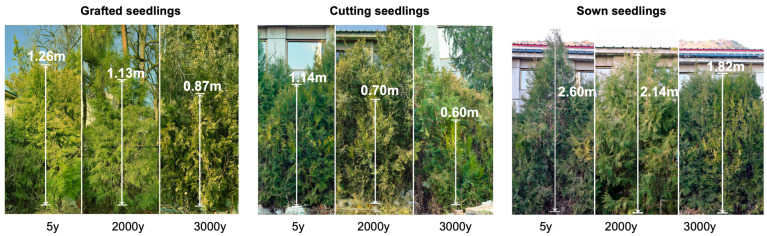
Sample trees were 5-year-old uniform seedlings of *P. orientalis* propagated from donors at different ages (5-, 2000-, and 3000-year-old) in different methods (seed sowing, cutting, and grafting), respectively.

**Figure 2:**
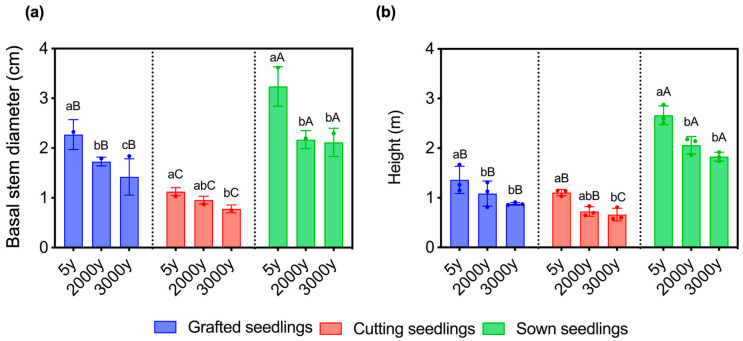
Variation in (**a**) basal stem diameters and (**b**) heights of seedlings of *P. orientalis* propagated from donors at different ages (5-, 2000-, and 3000-year-old) in different methods (seed sowing, cutting, and grafting), respectively. Letters (a–c) on top of the error bars show the significance of differences between seedlings propagated from donors at different ages (5-, 2000-, and 3000-year-old); Letters (A–C) on top of the error bars show the significance of differences between seedlings propagated in different propagation methods (seed sowing, cutting, and grafting). Values are the mean of three replicates of samples, and bars represent standard errors. Means with the different letters are significantly different at *p* < 0.05 by Duncan’s multiple range test.

The growth of 6-year-old *P. orientalis* seedlings was significantly affected by propagation methods and the age of the donors (Figure 1). In every age of the donors, basal stem diameter and plant height of the sown seedlings are the greatest, while those of cutting seedlings are the least. In every propagation method, basal stem diameter and plant height of the seedlings propagated from 5-year-old donors are the greatest, while those from 3000-year-old donors are the least, but there is no significant difference between those from 2000- and 3000-year-old donors (Figure 2). It suggests that the growth phenotype of seedlings was significantly affected by both the age of donors and propagation methods.

### 2.2. Age of Donors and Propagation Methods Affect the Soluble Sugar and Fatty Acid Contents of Seedlings in Winter

**Figure 3:**
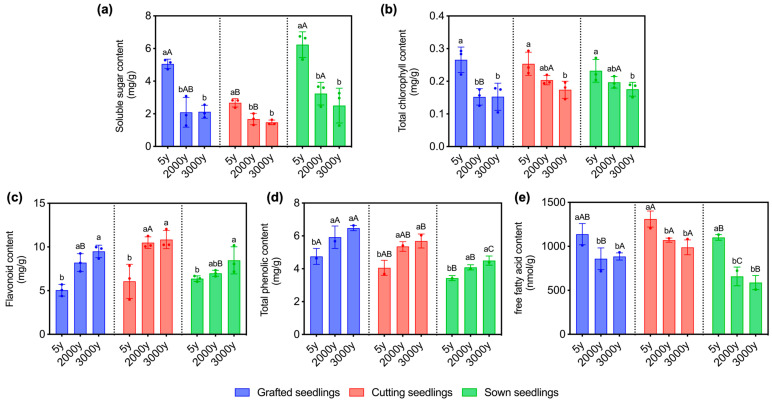
Effects of the contents of (**a**) soluble sugar, (**b**) total chlorophyll, (**c**) flavonoid, (**d**) total phenolic, and (**e**) free fatty acid in seedlings of *P. orientalis* propagated from donors at different ages (5-, 2000-, and 3000-year-old) in different methods (seed sowing, cutting, and grafting), respectively under low temperature. Letters (a,b) on top of the error bars show the significance of differences between seedlings propagated from donors at different ages (5-, 2000-, and 3000-year-old); Letters (A–C) on top of the error bars show the significance of differences between seedlings propagated in different propagation methods (grafting, cutting and seed sowing). Values are the mean of three replicates of samples, and bars represent standard errors. Means with the different letters are significantly different at *p* < 0.05 by Duncan’s multiple range test.

In every propagation method, the contents of soluble sugar, total chlorophyll, and free fatty acid of the seedlings propagated from 5-year-old donors are the greatest, while those from 3000-year-old donors are the least except for the contents of soluble sugar and free fatty acid of the grafted seedlings. There is no significant difference between the contents of soluble sugar and free fatty acid of the grafted seedlings propagated from 2000-year-old donors and that from 3000-year-old ones (Figure 3a,b,e). The flavonoid and total phenolic contents of 5-year-old donor’s propagated seedlings were significantly lower than those of 2000-year-old and 3000-year-old donor’s propagated seedlings (Figure 3c, d). The results showed that the age of donors and propagation methods significantly influenced the physiological and biochemical characteristics of the winter *P. orientalis* seedlings.

### 2.3. Age of Donors and Propagation Methods Affect the Expression of Genes Related to Phenylpropanoid Biosynthesis and Fatty Acid Metabolism of Seedlings in Winter

The apical leaves of the selected 6-year-old *P. orientalis* seedlings from 5-, 2000-, and 3000-year-old donors with 3 propagation methods in winter. After sequencing, a total of 156,177,968 reads were obtained and assembled into 204,798 unigenes. The average length of the assembled unigenes was 762.6 bp. The value of N50 was 1428 bp, and the GC percent value was 38.68%. Assessment of assembly integrity was conducted using BUSCO and scored C: 85.6%. The number of sequences with lengths 300–500 bp and 500–1000 bp was 56,732 and 36,205, respectively. There were 19,647 unigenes of 1000–2000 bp and 17,579 unigenes of >2000 bp. Most unigenes were in the range of <300 bp, with an abundance of 36% (Table 1), indicating that the sequencing data met the strict quality requirements for subsequent transcriptome analysis.

The unigenes of *P. orientalis* were annotated in the NR, NT, KO, Swiss Prot, PfamKOG, GO, and COG databases, with a success rate of unigene annotation of 100%. All the 204,798 unigenes of *P. orientalis* could be matched in the database (Table 2).

**Figure 4:**
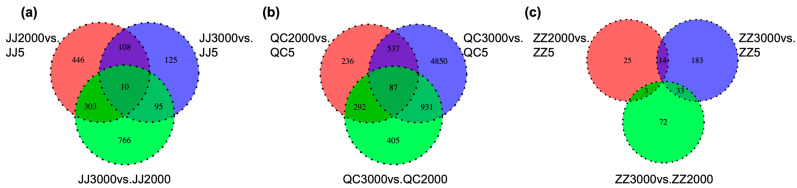
Venn diagram of differentially expressed genes (DEGs) in different comparisons. All DEGs are grouped into three comparison groups represented by three circles. The overlapping portions of the different circles represent the numbers of DEGs common to these comparison groups. (**a**) Venn diagram of grafted seedlings propagated from donors at different ages (5-, 2000-, and 3000-year-old) (**b**) Venn diagram of cutting seedlings propagated from donors at different ages (5-, 2000-, and 3000-year-old). (**c**) Venn diagram of sown seedlings propagated from donors at different ages (5-, 2000-, and 3000-year-old).

In every group of seedlings propagated in the same method, differences were investigated at the molecular level between the seedlings propagated from donors at different ages. The number of DEGs in the comparison groups of cutting seedlings was the largest, while that of sown seedlings was the least (Figure 4). The comparison group of cutting seedlings, 3000- vs. 5-year-old donors, has the most DEGs (4850), followed by 766 DEGs in the comparison group of grafted seedlings, 3000- vs. 2000-year-old donors, and 446 DEGs in the comparison group of sown seedlings, 2000- vs. 5-year-old donors. Therefore, donor trees of different ages have the greatest effect on cutting seedlings, but the least effect on sown seedlings.

**Figure 5:**
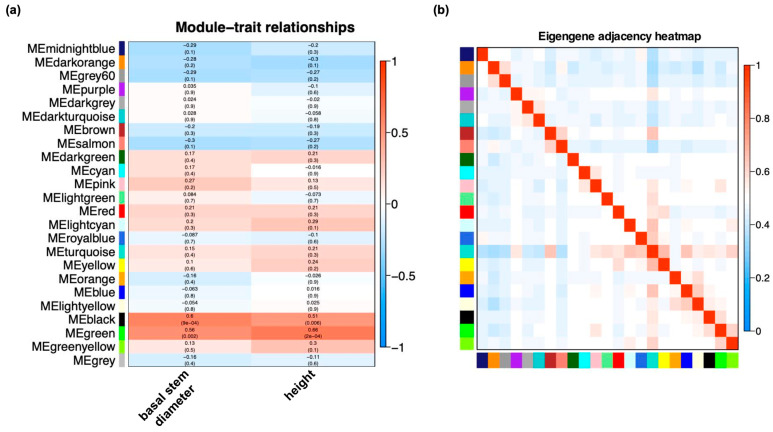
WGCNA revealed the modules highly related to basal stem diameters or heights. (**a**) Module–trait relationship. Each row corresponds to a module, while each column corresponds to the growth trait. Each cell contains a corresponding correlation. (**b**) The heatmap of connectivity of eigengenes.

In total, 22,584 DEGs were obtained in all comparison groups of seedlings propagated from donors at different ages in different propagation methods. Through WGCNA (weighted gene co-expression network analysis), 24 modules were identified. The black and green modules were significantly positively correlated with the growth of seedlings (the black module contained 634 DEGs, and the green module contained 1017 DEGs; the correlation coefficients of black and green modules with diameter were r = 0.6 and r = 0.56, respectively; the correlation coefficients of black and green modules with height were r = 0.51 and r = 0.66, respectively) (Figure 5).

**Figure 6:**
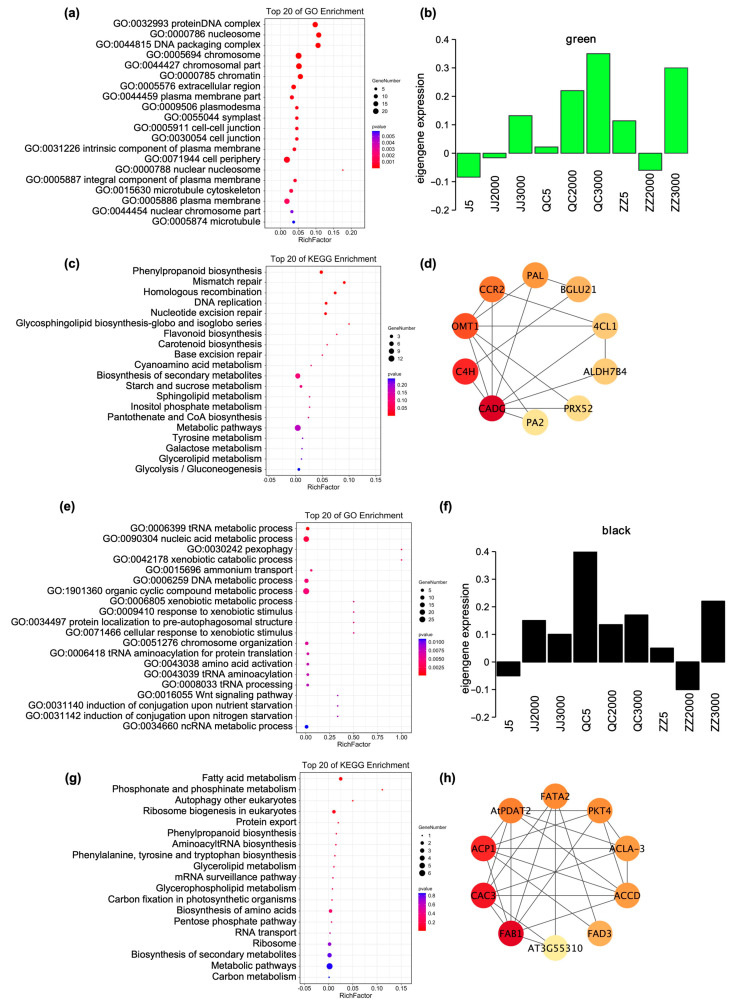
(**a**) Gene ontology and (**c**) Kyoto Encyclopedia of Genes and Genomes (KEGG) enrichment analyses of module green. (**e**) Gene ontology and (**g**) Kyoto Encyclopedia of Genes and Genomes (KEGG) enrichment analyses of module black; Expression profile and transcriptional regulatory network associated with the tissue-specific modules. (**b**) Module green and (**f**) module black show the consensus expression pattern of the co-expressed genes in each module. Note: JJ5: grafted seedlings propagated from 5-year-old donors, JJ2000: grafted seedlings propagated from 2000-year-old donors, JJ3000: grafted seedlings propagated from 3000-year-old donors; QC5: cutting seedlings propagated from 5-year-old donors, QC2000: cutting seedlings propagated from 2000-year-old donors, QC3000: cutting seedlings propagated from 3000-year-old donors; ZZ5: sown seedlings propagated from 5-year-old donors, ZZ2000: sown seedlings propagated from 2000-year-old donors, and ZZ3000: sown seedlings propagated from 3000-year-old donors. (**d**,**h**) represent the hub genes in transcriptional regulatory network of module green and module black, respectively. The color from dark to light indicates the Betweenness Centrality value from large to small.

Enriched biological processes in the green module included “chromosome” and “cell periphery” (Figure 6a), indicating that the genes may be involved in the mechanism of maintaining cell polarity during the recovery after environmental stresses or disease resistance through intracellular chromosomes and the periphery of plant cells. The pathways enriched in the green module included metabolic pathways, biosynthesis of secondary metabolites, and phenylpropanoid biosynthesis (Figure 6c), and the genes showed the highest expression levels to improve cold resistance in the cutting seedlings of propagated from 3000-year-old donors (Figure 6b). The results show that the expression levels of phenylpropion-related genes in cutting seedlings propagated from ancient trees were higher than that of sown seedlings and grafted seedlings.

GO (Gene Ontology) enrichment classification of the black module revealed that the nucleic acid metabolic process was most significantly enriched (Figure 6e), indicating that the genes in the black module may be involved in the nucleic acid synthesis and metabolism in plant cells. The KEGG (Kyoto Encyclopedia of Genes and Genomes) enrichment analysis of black module genes also revealed that fatty acid metabolism was the most significantly enriched metabolic pathway among various pathways (Figure 6g). The gene expression levels in cutting seedlings were significantly higher than that of grafted seedlings and sown seedlings (Figure 6f), indicating that the genes in this module are mainly involved in the fatty acid metabolic pathway under low-temperature stress. Therefore, it suggests that the cutting seedlings are more resistant to low temperatures than grafted seedlings and sown seedlings.

### 2.4. Identification of Hub Genes of Co-Expressed Modules

**Figure 7:**
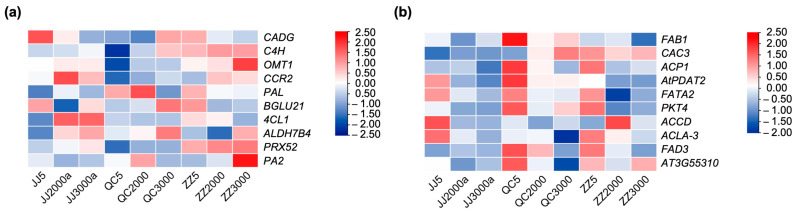
The changes in expression levels of the hub genes of module green (**a**) and module black (**b**) in *P.orientalis* seedlings propagated from donors at different ages (5-, 2000-, and 3000-year-old) in different propagation methods (grafting, cutting, and seed sowing). Note: JJ5: grafted seedlings propagated from 5-year-old donors, JJ2000: grafted seedlings propagated from 2000-year-old donors, JJ3000: grafted seedlings propagated from 3000-year-old donors; QC5: cutting seedlings propagated from 5-year-old donors, QC2000: cutting seedlings propagated from 2000-year-old donors, QC3000: cutting seedlings propagated from 3000-year-old donors; ZZ5: sown seedlings propagated from 5-year-old donors, ZZ2000: sown seedlings propagated from 2000-year-old donors, and ZZ3000: sown seedlings propagated from 3000-year-old donors.

To identify the hub genes in module green and module black, a modular hub gene transcriptional regulatory network was constructed by CytoNCA according to the Betweenness Centrality ranking of genes (Figure 6d,h, Supplementary Table S1). Ten hub genes related to the phenylpropanoid biosynthesis pathway were identified in module green (Supplementary Table S2). Among them, the expression levels of *C4H*, Flavone 3′-O-methyltransferase 1 (*OMT1*), Dihydroflavonol-4-reductase (*CCR2*), *PAL*, and Peroxidase 52 (*PRX52*) were increased in old *P. orientalis* donor seedlings (Figure 7a). It suggests that phenylpropanoid biosynthesis played a key role in the resistance of seedlings propagated from old donors to low-temperature stress.

Ten hub genes were identified in the black module (Supplementary Table S2). Fatty acid metabolism-related genes Acyl carrier protein 1 (*ACP1*), Diacylglycerol acyltransferase 2 (*AtPDAT2*), and Omega-3 fatty acid desaturase (*FAD3*) were highly expressed in 5-year-old cutting seedlings (Figure 7b). *ACP1* is an important member of plant fatty acid biosynthesis; *ACP* catalyzed the insertion of a double bond into saturated fatty acid bound in saturated acyl chains bound to *ACP* in plants, producing cis-monounsaturated fatty acids [21]. This indicated that the expression of the genes related to fatty acid metabolism and abiotic stress response is greatly affected by the propagation methods. It suggests that cutting seedlings have greater ability in plant fatty acid accumulation to improve their defense capability.

### 2.5. qRT-PCR Validation of Gene Expression Profiles of DEGs

**Figure 8:**
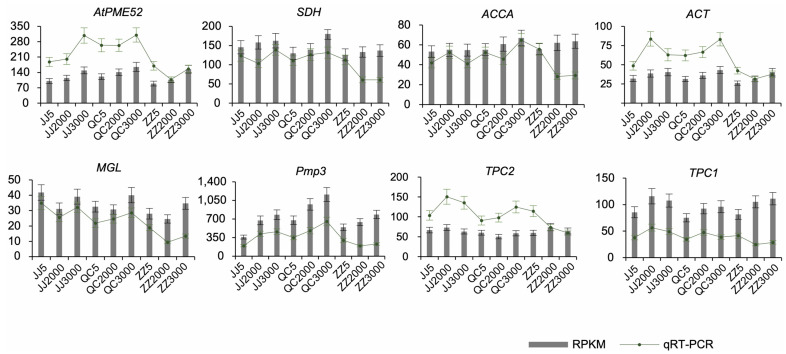
qRT-PCR analysis of seedlings of *P. orientalis* propagated in different methods from donors at different ages under low-temperature stress. Data are from three biological replicates and three technical replicates. The differential expression analysis was conducted based on the 2^−ΔΔct^ method. Note: JJ5: grafted seedlings propagated from 5-year-old donors, JJ2000: grafted seedlings propagated from 2000-year-old donors, JJ3000: grafted seedlings propagated from 3000-year-old donors; QC5: cutting seedlings propagated from 5-year-old donors, QC2000: cutting seedlings propagated from 2000-year-old donors, QC3000: cutting seedlings propagated from 3000-year-old donors; ZZ5: sown seedlings propagated from 5-year-old donors, ZZ2000: sown seedlings propagated from 2000-year-old donors, and ZZ3000: sown seedlings propagated from 3000-year-old donors.

To verify the accuracy of the RNA-Seq data, eight unigenes highly expressed under low-temperature stress were selected, and their expression levels were determined by qRT-PCR with three biological replicates. The primers for the eight selected unigenes are listed in Supplementary Table S3. The expression levels of the unigenes vary in seedlings propagated from donors at different ages in different propagation methods, showing that the transcriptional profiles are generally reflected by the RNA-Seq data (Figure 8). Therefore, our RNA-seq and qRT-PCR analysis coincide, showing the results are reliable to advance further research.

## 3. Discussion

**Figure 9:**
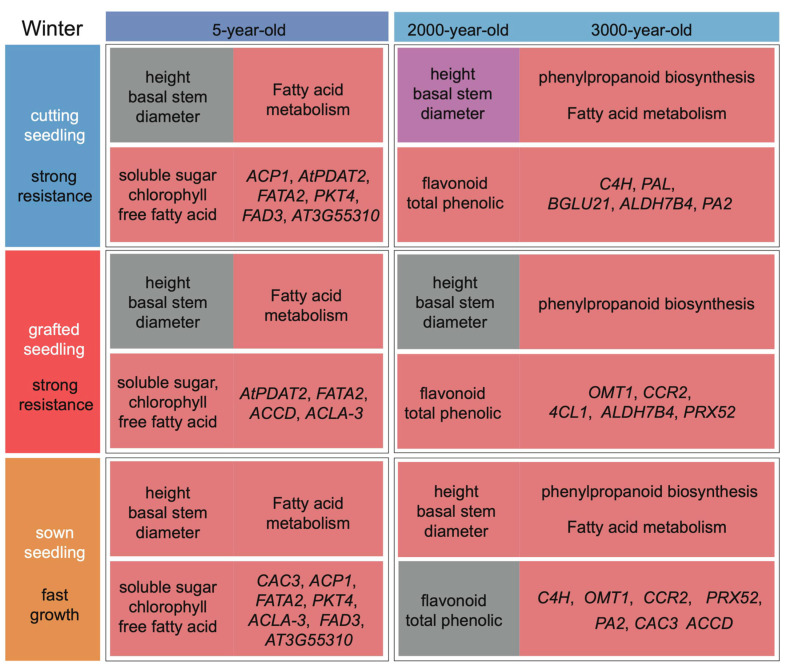
Framework plots of growth indicators, physiological indicators, and molecular expression levels of *P. orientalis* propagated from donors at different ages (5-, 2000-, and 3000-year-old) in different methods (seed sowing, cutting, and grafting). Red represents high values, grey represents medium values, and purple represents low values.

Cold tolerance in seedlings of *P. orientalis* was affected by the age of donors and the propagation methods. Our study showed that sown seedlings of *P. orientalis* grew significantly faster than cut seedlings and grafted seedlings under low temperatures. The results are consistent with those in *Medicago sativa* seedlings [22]. Grafted tomato seedlings grow better at low temperatures than sown seedlings, which may be caused by the fact that the rootstocks for grafting can provide additional nutrients and hormones [23]. The age of the donors is another important factor affecting the cold tolerance of the seedlings propagated in different methods. During the process of low-temperature stress, the cold tolerance of older *Thinopyrum intermedium* was enhanced [24]. In this study, comparative phenotypic, physiological, and transcriptomic analyses of different seedlings propagated from donors at different ages in different propagation methods showed that the ability of seedlings to tolerate the cold is strongly influenced by the age of the donors. Based on the collective results of this study, we constructed framework plots of growth indicators, physiological indicators, and molecular expression levels of *P. orientalis* propagated from donors at different ages (5-, 2000-, and 3000-year-old) in different methods (seed sowing, cutting, and grafting) (Figure 9).

Propagation methods and donor age affected physiological changes in *P. orientalis* seedlings under low-temperature stress. Singh et al. suggested that the increased contents of chlorophyll and soluble sugar were indicators of cold tolerance in maize [25]. This study found that the contents of soluble sugar and chlorophyll were high in 5-year-old *P. orientalis* seedlings and low in 2000- and 3000-year-old seedlings under low-temperature conditions, and the highest value of the content of soluble sugar was found in sown seedlings of the same age of donors. The cutting seedlings of *Robinia pseudoacacia* propagated from 2-year-old donors grew better than that from 15-year-old ones [26]. The soluble sugars and chlorophyll contents of *P. tremuloides* and *Pinus nigra* decreased with increasing plant ages [27,28], respectively. This is consistent with the results of this present study. This reflects to some extent the more active life activities such as photosynthesis, metabolism, and transformation of substances in young donor *P. orientalis* leaves.

Phenylpropanoid biosynthesis is a major secondary metabolic process in plants, which produces lignin, flavonoids, and so on [29]. Phenolic and flavonoid compounds contribute significantly to plant defense mechanisms [24]. In this study, the flavonoid and total phenolic contents of seedlings propagated from 2000- and 3000-year-old donors with different propagation methods were higher than those from 5-year-old ones, and those of sown seedlings were lower than those of grafted seedlings and cut seedlings. Interestingly, our transcriptome results showed that the expression levels of DEGs in this pathway were the highest in cutting seedlings propagated from 3000-year-old donors, and the expression levels of hub genes *C4H*, *OMT1*, *CCR2*, *PAL*, and *PRX52* were increased in old *P. orientalis* propagation seedlings, while cold stress induced the expression of structural genes in the phenylpropanoid pathway, including *CHS* and 4-coumarate:coenzyme A ligase (*4CL*) [30], which is in line with the research results in *Phyllostachys edulis* [31] and *Morus nigra* [32]. This is due to the lignification of stems, thickening of cell walls, and accumulation of lignin with increasing donor age to improve plant defenses [12,13]. It is consistent with the results in *P. orientalis* that the highest content of secondary metabolites was detected in seedlings propagated from 3000-year-old donors. However, a study by Xu et al. found that phenylpropanoid and flavonoid biosynthesis was significantly up-regulated in grafted *Carya cathayensis* [33]. This may be related to increased contents of a series of downstream antioxidant secondary metabolites formed in flavonoid biosynthesis in grafted seedlings. Therefore, the differences in secondary metabolites in the seedlings propagated in different methods under cold stress need to be further studied.

Under low-temperature stress, unsaturated fatty acids in plants are beneficial to maintain the stability of cell membranes [15,34]. In this study, the content of free fatty acid in seedlings propagated from 5-year-old *P. orientalis* donors was higher than that from 2000- and 3000-year-old ones, and the content was highest in cutting seedlings and least in sown seedlings. Similarly, the expression level of hub genes *ACP1*, *AtPDAT2*, and *FAD3* was highly expressed in 5-year-old cutting seedlings. The transcript and protein levels of *ACP* are affected by various biotic and abiotic stresses [35,36]. So far, no studies on the *ACP1* gene in *P. orientalis* have been reported, and it could be taken seriously in further studies. *PDAT* catalyzes and synthesizes triacylglycerol (TAG) and participates in oil accumulation and abiotic stress response [37,38]. Overexpression of *FAD3* promotes jasmonate biosynthesis and enhances plant cold tolerance in *Arabidopsis thaliana* [39]. It is also concluded that the levels of fatty acids decrease in *A. thaliana*, *Brachypodium distachyon*, and *Panicum virgatum* with the age increasing [40]. The increased unsaturated fatty acids in cutting seedlings of hybrid poplar improved survival under freezing stress [41], which is consistent with our current findings. It suggests that the plants propagated from young donors are more resistant to low temperatures than those from ancient trees, and cutting propagation is the best way to maintain the ability to tolerate the low temperature. Although there have been comparative studies on leaves of seedlings of different ages, the dynamics of leaf physiological indicators and resistance to low-temperature stress with donor age still need to be investigated in depth in conjunction with leaf anatomy, morphology, and epigenetics.

## 4. Materials and Methods

### 4.1. Plant Materials and Growth Conditions

In the experimental nursery located at the main campus of Chinese Academy of Forestry, Haidian District, Beijing, China (40°0′39.40″ N, 116°15′15.35″ E), field experiments were conducted during 2013–2020.

**Figure 10:**
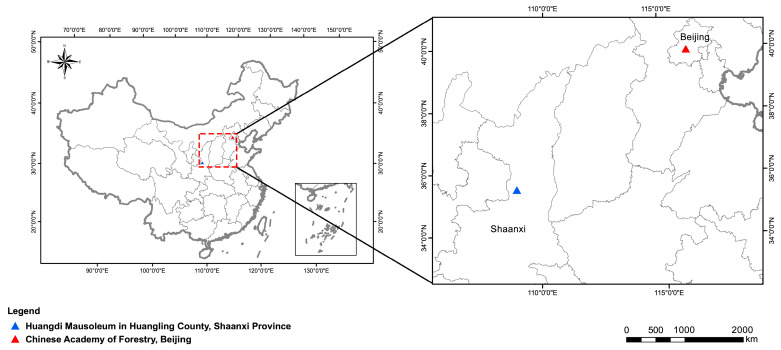
The location of the sampling point distribution area. The different ages (5-, 2000-, and 3000-year-old) *P. orientalis* for seed sowing, cutting, and grafting were collected from Huangling County, Shaanxi Province, China (35°58′52.84″ N, 119°27′12.27″ E). Field experiments were located at the experimental nursery on the main campus of the Chinese Academy of Forestry, Haidian District, Beijing, China (40°0′39.40″ N, 116°15′15.35″ E).

*P. orientalis* trees of different ages (5-, 2000-, and 3000-year-old) located in Huangling County, Shaanxi Province, China (35°58′52.84″ N, 119°27′12.27″ E), were used to provide seeds for sowing, current-year stems with a diameter of 2.65 mm for cutting and current-year-branches as scions. Sowing was performed on 5 April 2014. Stem cuttings were planted on 5 June 2015. Grafting was performed with 2-year-old sown seedlings with a ground diameter of 1.7 cm as rootstocks by using the side grafting method on 5 June 2015 (Figure 10). All seedlings were grown in pots (40 cm in diameter × 38.5 cm in height) containing soil mixture consisting of garden soil, peat, and vermiculite (2/1/1 *w*/*w*/*w*). They were watered every two weeks in spring, summer, and autumn, while only once during the winter season (December–February). The distance between adjacent pots was 40 cm. They were fertilized with slow-release fertilizer (15-15-15 NPK) at a rate of 1 g per pot every 3 months. In mid-December 2020 (average temperature was −6 °C–−3 °C), the uniform seedlings of *P. orientalis* propagated from donors at different ages (5-, 2000-, and 3000-year-old) in different methods (seed sowing, cutting, and grafting), respectively, were selected for experimentation.

The apical leaves of the selected 6-year-old seedlings were harvested in winter (Figure 1). Phenotype, physiological, and transcriptomic analyses were performed with three biological replicates per experiment. The collected apical leaves were immediately frozen in liquid nitrogen and then kept at −80 °C until total RNA extraction.

### 4.2. Phenotypic Measurements

Experiments were performed in a completely randomized manner. Basal stem diameter was measured at 2 cm above the ground with a digital caliper. Plant height was measured from the base of the main stem to the tip of the highest leaf with a tape measure.

### 4.3. Determination of Soluble Sugar and Chlorophyll Contents

About 0.2 g of apical leaves were used to measure the content of soluble sugar. The anthrone colorimetric method was used to determine the content of soluble sugar based on absorbance at 620 nm [42]. About 0.1 g of apical leaves were well grounded under dark conditions, and chlorophyll was extracted with acetone and 90% ethanol (*v*/*v*) at 4 °C under weak light for 3 h until the apical leaves turned blanch. Chlorophyll a, chlorophyll b, and total chlorophyll contents were calculated based on the enzyme-labeled instrument’s absorbance readings at 663 nm and 645 nm.

### 4.4. Determination of Flavonoids, Total Phenolics and Fatty Acids

Total flavonoids were estimated according to the aluminum chloride method [43]. Briefly, 0.02 g of ground sample was extracted with 60% ethanol (*v*/*v*) shaking at 60 °C for 2 h. A mixture of 108 µL of supernatant, 6 µL of sodium nitrite (NaNO_2_) (1:20 *w*/*v*), and 6 μL of aluminum chloride (AlCl_3_) (1:10 *w*/*v*) was incubated at 25 °C for 6 min. Then, 1 N sodium hydroxide (80 µL) was added to the mixture. It was mixed thoroughly using a vortex device. The absorbance was measured at 510 nm. A blank was prepared following the same procedure using distilled water. Rutin concentrations ranging from 0 to 1200 µg/mL were prepared, and the standard calibration curve was obtained using a linear fit (r^2^ = 0.9980). The concentration of flavonoids was determined from the absorbance.

The total phenolics content was determined by Folin–Ciocalteu colorimetric method [44]. Briefly, 0.1 g of ground sample was started with 60% ethanol (*v*/*v*) at 60 °C. Then, 10 µL of sample and 50 µL of Folin–Ciocalteu reagent was added. The contents were vortexed for 10 s and then kept at room temperature for 2 min. Additionally, 50 µL of 5% (*w*/*v*) sodium carbonate solution was added to stop the reaction, then 90 µL of distilled water was added to make up to 200 µL. The reaction mixture was incubated at 25 °C for 10 min and absorbance was measured at 760 nm. Gallic acid concentrations ranging from 0 to 300 µg/mL were prepared, and the calibration curve was obtained using a linear fit (r^2^ = 0.9961).

The colorimetric determination of fatty acids was performed using the copper soap method described by Veerapagu et al. [45]. Under weakly acidic conditions, FFA reacted with copper salts to produce copper soap with a characteristic absorption peak at 715 nm and a linear relationship between the free fatty acid content and the degree of color development within a certain range. A total of 0.1 g of sample was started by shaking with 1.0 mL isooctane for 2 h. Aliquots of 0.4 mL of the upper layer were pipetted into a test tube and then 0.2 mL of cupric acetate-pyridine reagent was added. The solution was shaken well and incubated for 5 min, and then the absorbance was measured at 715 nm. A control tube was prepared using sodium acetate-pyridine solution following the same procedure. Oleic acid was used as the standard curve, and the free fatty acid content was determined by the copper-soap method.

### 4.5. RNA Sequencing Analysis

Total RNA was extracted using the RNAprep Pure Plant Kit (Tiangen, Beijing, China). The quality of the RNA was assessed using the NanoDrop 2000 spectrophotometer (Thermo Scientific, DE, USA). High-quality RNA was used to construct cDNA libraries, which were sequenced using the Illumina NovaSeq 6000 platform to generate paired-end reads. Clean reads were obtained by removing adapter sequences and low-quality reads were aligned to the reference genome using the TopHat2 software (v2.0.7) [46]. Clean reads were also mapped to the de novo *P. orientalis* transcriptome assembly using Cufflinks [47]. The protein-coding genes were annotated using the NCBI NR, SWISS-PROT, GO, COG, KOG, eggNOG, and KEGG databases. The expression level of each gene was estimated by fragments per kilobase of transcript per million mapped reads (FPKM). DEGs were analyzed using the R package DESeq2 (V 1.32.0) [48]. Important DEGs were identified according to the following criteria: each paired comparison; fold-change; ≥ 2; and FDR ≤ 0.001. The number of DEGs in different comparisons was visualized using the R package UpSetR (V 1.4.0) [49]. The weighted gene co-expression network was constructed through the WGCNA (V 1.70.3) package in R software [50]. Soft thresholds were calculated using the pick soft threshold function provided by WGCNA. Histograms were drawn using GraphPad Prism, and the gene cluster heat map was drawn using Tbtools [51].

### 4.6. Quantitative Real-Time PCR Analysis

Eight hub genes were selected for qRT-PCR validation. Total RNA was reversely transcribed into cDNA by using Prime Script™ Reagent Kit with gDNA Eraser (Takara, Dalian, China). The qRT-PCR assay was performed with KAPA SYBR FAST qPCR Master Mix (Kapa Bio-systems, USA), according to the instructions of the manufacturer. The primers used for qRT-PCR were designed using Premier-BLAST [52] and listed in Supplementary Table S1. The thermal cycling conditions were as follows: 95 °C for 30 s, 40 cycles at 95 °C for 5 s, and 60 °C for 30 s. *Actin* was used as the reference gene [53], and the relative expression values were calculated by using the 2^−ΔΔct^ method [54].

### 4.7. Statistical Analysis

The significance of the differences among the means of growth and physiological values were calculated using one-way analysis of variance (ANOVA), and the results were expressed as the mean ± standard error (SE), followed by Duncan’s test at *p* < 0.05. All statistical analyses were carried out by SPSS 22.0 (SPSS Inc., Chicago, IL, USA).

## 5. Conclusions

This study combined growth, physiological indicators, and transcriptome sequencing to comprehensively analyze the cold resistance mechanisms of seedlings of *P. orientalis* propagated from donor trees at different ages in different propagation methods. It was demonstrated that the growth, stress resistance, and physiological and biochemical characteristics of *P. orientalis* seedlings were strongly influenced by the ages of the donor trees. Transcriptome data demonstrated that phenylpropanoid biosynthesis and fatty acid biosynthesis pathways were the most enriched biological process in *P. orientalis* seedlings propagated from donor trees at different ages in different propagation methods under low temperature. It is worth mentioning that the hub gene *C4H*, *OMT1*, *CCR2*, *PAL*, *PRX52*, and *ACP1* were also found. It suggests that the plants propagated from young donors are more resistant to low temperatures than those from ancient trees. In addition, the ability to cut seedlings to maintain the resistance characteristics of ancient trees also indicates the reliability and stability of asexual propagation. Our findings provide evaluations of the seedlings from ancient *P. orientalis* propagated in different methods and ages of donors to improve the overall levels of *P. orientalis* clonal forestry.

There are various ways for plants to resist adversity, and under low-temperature stress, plants will adapt to a low-temperature environment through various physiological and biochemical changes for better growth. This experiment only measured morphological changes and some of the physiological and biochemical indicators under low-temperature stress, and if we want to study the cold resistance of seedlings in depth, we need to combine the photosynthetic characteristics of seedlings and other physiological indicators to conduct a comprehensive analysis. In addition, it is necessary to refine the temperature gradient settings to truly understand the effects of the propagation method and tree age on seedling cold resistance.

## Figures and Tables

**Table 1:** De novo assembly of the *P. orientalis* transcriptome.

Items	Number
Total unigenes	204,798
Total base	156,177,968
Average length (bp)	762.6
N50	1428
GC percent	38.68%
BUSCO score	C: 85.6%
Number of transcripts <300 bp	74,617 (36%)
Number of transcripts between 300 and 500 bp	56,732 (28%)
Number of transcripts between 500 and 1000 bp	36,205 (18%)
Number of transcripts between 1000 and 2000 bp	19,647 (10%)
Number of transcripts >2000 bp	17,597 (9%)

**Table 2:** Functional annotation of the *P. orientalis* transcriptome.

Database	Number of Unigenes	Percentage (%)
Annotated in NR	62,859	30.69%
Annotated in NT	28,559	13.94%
Annotated in KO	10,136	4.95%
Annotated in SwissProt	41,489	20.26%
Annotated in Pfam	59,635	29.12%
Annotated in GO	34,306	16.75%
Annotated in COG/KOG	34,431	16.81%
Annotated in all Databases	3262	1.59%
Annotated in at least one Database	79,514	38.83%
Total Unigenes	204,798	100%
Annotated in NR	62,859	30.69%

## Data Availability

The data presented in this study are available in Supplementary Material.

## Supplementary Materials

The following supporting information can be downloaded at: https://www.mdpi.com/article/10.3390/ijms24087170/s1.
