# Supplementary material for: Effects of Donor Ages and Propagation Methods on Seedling Growth of Platycladus orientalis (L.) Franco in Winter
Source: Int J Mol Sci. 2023 Apr 12;24(8):7170. doi: 10.3390/ijms24087170 (PMC10138323; doi:10.3390/ijms24087170)
Supplement: Supplementary file 1 [file ijms-24-07170-s001.zip › ijms-2324800-supplementary.pdf]

Supplementary Table S1. Betweenness Centrality value ranking of hub genes.

Modules green		Modules black	
GeneID	Score	GeneID	Score
CADG	314.67	*FAB1*	21.53
C4H	184.33	*CAC3*	20.67
OMT1	67.33	*ACP1*	19.90
CCR2	44.0	*AtPDAT2*	14.13
PAL	25.67	*FATA2*	13.33
BGLU21	19.0	*PKT4*	12.90
4CL1	14.33	*ACCD*	12.03
ALDH7B4	14.33	*ACLA-3*	12.03
PRX52	11.53	*FAD3*	10.67
PA2	8.0	*AT3G55310*	6.0

Supplementary Table S2. Hub genes detected in the green and black modules.

Modules	Gene_id	Gene annotation
green	TRINITY_DN52222_c0_g3	*CADG* (*Probable cinnamyl alcohol dehydrogenase*)
	TRINITY_DN57476_c1_g2	*C4H* (*Cinnamate 4-hydroxylase*)
	TRINITY_DN55807_c0_g1	*OMT1* (*Flavone 3'-O-methyltransferase 1)*
	TRINITY_DN50497_c0_g2	*CCR2* (*Dihydroflavonol-4-reductase*)
	TRINITY_DN2977_c0_g1	*PAL* (*Phenylalanine ammonialyase*)
	TRINITY_DN54540_c4_g1	*BGLU21* (*Beta-glucosidase 21*)
	TRINITY_DN48909_c0_g1	*4CL1* (*4-coumarate--CoA ligase 1*)
	TRINITY_DN54466_c2_g5	*ALDH7B4* (*Aldehyde dehydrogenase*)
	TRINITY_DN55621_c3_g5	*PRX52* (*Peroxidase 52*)
	TRINITY_DN39540_c0_g1	*PA2* (*Peroxidase 53*)
black	TRINITY_DN44444_c0_g1	*FAB1* (*3-oxoacyl-[acyl-carrier-protein] synthase 2*)
	TRINITY_DN49634_c1_g1	*CAC3* (*Acetyl-coenzyme A carboxylase carboxyl transferase subunit alpha*)
	TRINITY_DN40677_c0_g1	*ACP1*(*Acyl carrier protein 1*)
	TRINITY_DN140057_c0_g	*AtPDAT2* (*Putative phospholipid:diacylglycerol acyltransferase 2*)
	TRINITY_DN4115_c0_g1	*FATA2* (*Oleoyl-acyl carrier protein thioesterase 2*)
	TRINITY_DN63470_c0_g1	*PKT4* (*3-ketoacyl-CoA thiolase 1*)
	TRINITY_DN55731_c2_g3	*ACCD* (*Acetyl-coenzyme A carboxylase carboxyl transferase subunit beta*)
	TRINITY_DN72476_c0_g1	*ACLA-3* (*Elongation of fatty acids protein 3*)
	TRINITY_DN44771_c0_g1	*FAD3* (*Omega-3 fatty acid desaturase*)
	TRINITY_DN99232_c0_g1	*AT3G55310* (*Elongation of fatty acids protein A*)

Supplementary Table S3 Primers of the candidate genes designed for qRT-PCR

Gene ID	Gene abbreviation	Gene name	Size (bp)	Forward primer<br>Reverse primer
TRINITY_DN53682_c1_g1	*AtPME52*	*Pectin methylesterase 52*	104	TGTTCGACAAGTGCAAGAGG<br>CACCTTGTCTCCTCCCAAAA
TRINITY_DN57178_c2_g1	*SDH*	*Sorbitol dehydrogenase*	92	CTTGGAAGGTTTGTGGAGGA<br>CATGGTGGCAGTTTTGTCTG
TRINITY_DN49634_c1_g1	*ACCase*	*Acetyl-CoA carboxylase 1*	107	TCCCATACAATCCCACCAAG<br>CGCCTAGCTCAATGGTTGAT
TRINITY_DN52424_c0_g1	*ACT*	*Agmatine coumaroyltransferase-2*	123	TATCGAACCGGAGCAGAATC<br>TGCGAGCTAAGAAAGGGAAA
TRINITY_DN44726_c0_g1	*MGL*	*Monoglyceride lipase*	101	GAGGTGCTTGGAGTCCTGAA<br>TTTCCACATCGAAGGAGACC
TRINITY_DN48651_c1_g2	*ESI3*	*Salt stress-induced hydrophobic peptide*	108	GCGGTATGGGTTGTAGTGCT<br>GTCGTTGCCGAAGTACCAAT
TRINITY_DN48518_c0_g1	*ACX*	*Acyl-CoA oxidase*	148	GCGCAAGTTTGGCTCTTTAC<br>AGCCCATGAATCATTTGAGC
TRINITY_DN55657_c4_g1	*TPC1*	*Two pore calcium channel protein 1*	147	CCAAAGGGCCATTTTGTTGT<br>CGTGGTTGAAGCCAAATTCT
